# Umbilical hernia rupture with evisceration of omentum from massive ascites: a case report

**DOI:** 10.1186/1752-1947-5-170

**Published:** 2011-05-03

**Authors:** Daniel W Good, Jonathan E Royds, Myles J Smith, Paul C Neary, Emmanuel Eguare

**Affiliations:** 1 Minimally Invasive Surgical Unit, Division of Colorectal Surgery, AMNCH, Tallaght, Dublin 24, Ireland

## Abstract

**Introduction:**

The incidence of hernias is increased in patients with alcoholic liver disease with ascites. To the best of our knowledge, this is the first report of an acute rise in intra-abdominal pressure from straining for stool as the cause of a ruptured umbilical hernia.

**Case presentation:**

An 81-year-old Caucasian man with a history of alcoholic liver disease presented to our emergency department with an erythematous umbilical hernia and clear, yellow discharge from the umbilicus. On straining for stool, after initial clinical assessment, our patient noted a gush of fluid and evisceration of omentum from the umbilical hernia. An urgent laparotomy was performed with excision of the umbilicus and devitalized omentum.

**Conclusion:**

We report the case of a patient with a history of alcoholic liver disease with ascites. Ascites causes a chronic increase in intra-abdominal pressure. A sudden increase in intra-abdominal pressure, such as coughing, vomiting, gastroscopy or, as in this case, straining for stool can cause rupture of an umbilical hernia. The presence of discoloration, ulceration or a rapid increase in size of the umbilical hernia signals impending rupture and should prompt the physician to reduce the intra-abdominal pressure.

## Introduction

The anterior abdominal wall has multiple areas of potential weakness (deep and superficial inguinal rings, Hesselbach's triangle, the femoral ring and so on) which, when exposed to acute or chronically elevated intra-abdominal pressure, are prone to weaken and allow the formation of various hernias [1]. The umbilicus is one of these areas of potential weakness as it interrupts the continuity of the linea alba [1].

Intra-abdominal pressure varies in both an acute and a chronic manner. During normal physiology acute variations in intra-abdominal pressure mainly follow changes in body position and patient activities [2-4]. In health subjects, causes of chronic increases in intra-abdominal pressure include obesity, visceromegaly and pregnancy [5,6]. Intra-abdominal pressure is also chronically elevated in various disease processes including ascites, large cysts and large neoplastic formations [7-9] which increase the likelihood of hernias.

## Case Presentation

**Figure 1:**
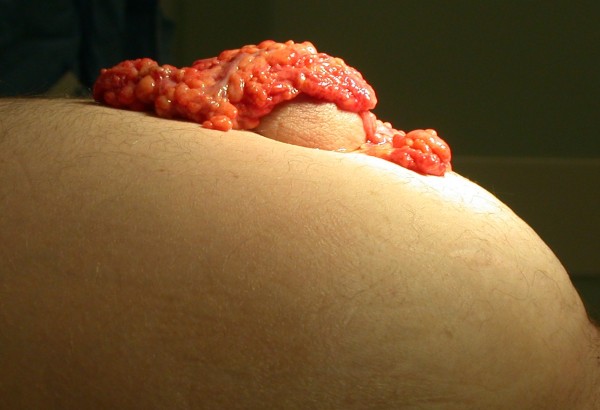
**Side on view of distended abdomen with an umbilical hernia with evisceration of omentum**.

**Figure 2:**
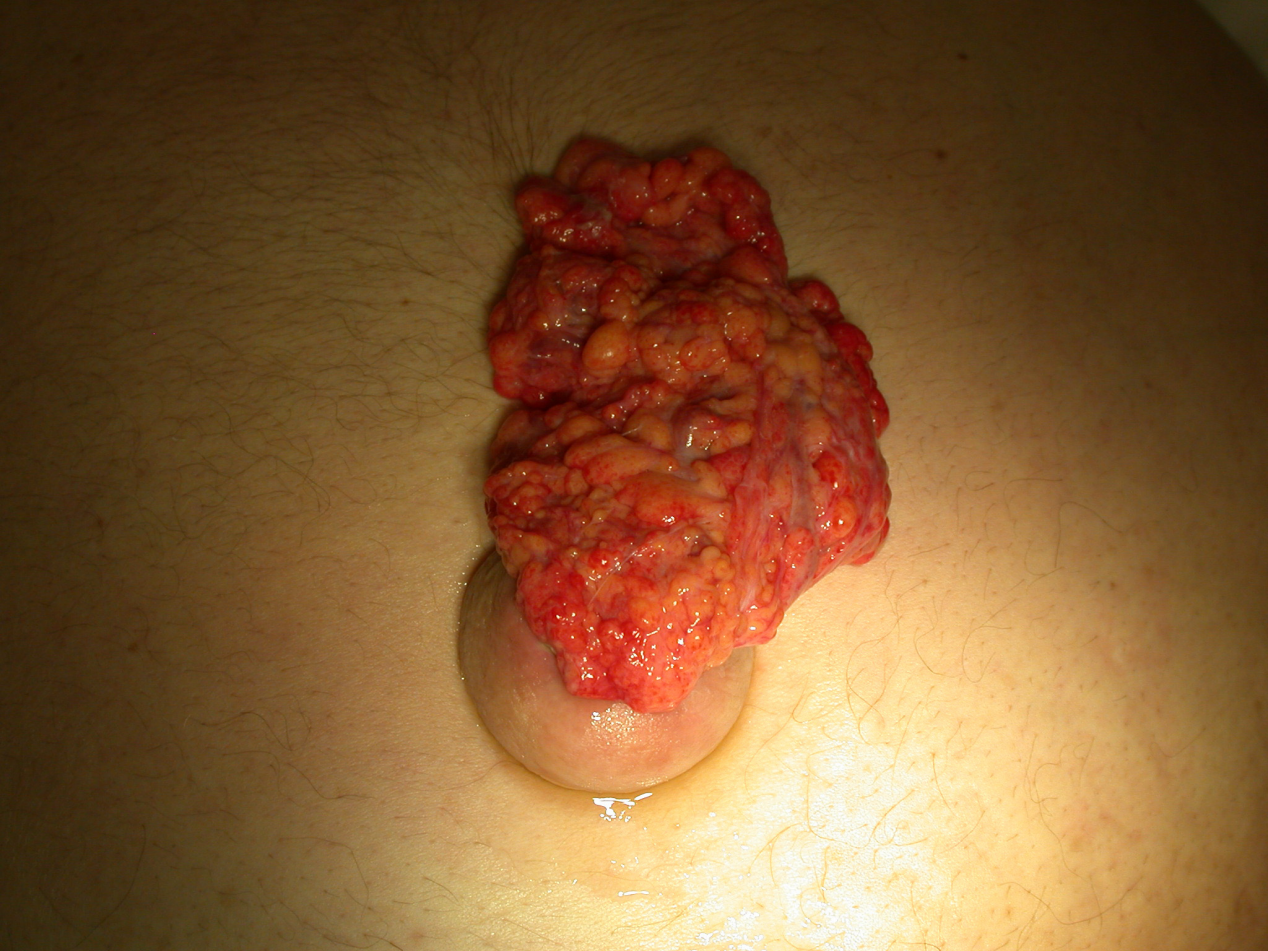
**Vertical view of distended abdomen with rupture of the umbilical hernia with evisceration of omentum**.

An 81-year-old Caucasian man, with a background history of alcoholic liver disease, presented acutely via our emergency department, with an erythematous umbilical hernia and clear, yellow discharge from the umbilicus. Clinical examination showed signs of decompensated liver disease, including asterixis, spider naevi, a distended abdomen with shifting dullness, fluid thrill and an erythematous umbilical hernia. On straining for stool, after initial clinical assessment, our patient noted a gush of fluid and evisceration of omentum from the umbilical hernia (Figures 1 and 2).

An urgent laparotomy was performed, using povidone-iodine solution for skin preparation via a midline incision, with excision of the umbilicus and devitalized omentum. Of note, there was evidence of recanalization of the umbilical vein. A full examination of the abdominal viscera was performed, and samples of ascitic fluid sent for cytological, biochemical and microbiological analysis. The liver was noted to be nodular, shrunken and sclerotic with generalized fibrinous exudate lining the coelomic cavity. His post-operative α-fetoprotein was 798 IU/mL. The abdominal fascial edges were re-apposed with interrupted 1/0 polypropylene sutures, with clips to the skin. The ascitic fluid serum-ascites albumin gradient was >1.1 g/dL, and showed increased ascitic protein level (>2.5 g/dl). Cytology was negative for malignant cells.

## Discussion

The incidence of hernias is increased in patients with alcoholic liver disease with ascites [10]. The first reported case of spontaneous rupture of an umbilical hernia from ascites was reported by Mixter in 1901 [11]. The precipitating factors for rupture described include local trauma and a sudden increase in intra-abdominal pressure, such as coughing, vomiting or esophagoscopy. To the best of our knowledge, straining for stool has not yet been reported in the literature as a cause of acute rupture of an umbilical hernia. All of the above precipitants are known to cause acute variations in the intra-abdominal pressure [3,4]. In the presence of chronic elevation of intra-abdominal pressure, such as occurs with ascites, these activities and patient positions cause an additional increase in intra-abdominal pressure which can overwhelm the strength of the anterior abdominal wall layers [12]. The presence of discolouration, ulceration or a rapid increase in size of the umbilical hernia signals impending rupture [13].

Current thinking suggests that there is a dynamic adaptive change which takes place in all organisms in response to a chronically elevated intra-abdominal pressure, principally as adaptations to the constitutional properties of the abdominal cavity. This occurs in order to maintain normal functioning [7,14-16]. These adaptations are mainly in the form of changes in muscular structures. There have been several animal studies showing that muscular components of the abdominal cavity, as well as the diaphragm, adapt when subjected to conditions of increasing intra-abdominal pressure [7,17]. However, it is likely that in more acute or sub-acute changes of intra-abdominal pressure, such as a sudden increase in ascites combined with straining for stool as in this case report, it may overcome the elasticity of the abdominal wall and lead to hernias or worse hernia rupture.

## Conclusion

There has been considerable debate in the literature as to the timing of umbilical hernia repair in patients with alcoholic liver disease and ascites. Older studies, in particular by Baron [18], described poor outcomes in elective repair with mortality rates of up to 38%. Some of the poor outcome was thought to involve a disruption of portal venous flow around the umbilicus, causing increased portal pressure which may lead to variceal bleeding. Other studies [19,20] have shown improved outcomes in the elective setting but require intensive pre-operative optimization. Some experts [21] would operate in the elective setting for Child's A cirrhosis and when complications of umbilical hernias develop an urgent repair is indicated. Current literature suggests that control of ascites post-operatively is critical to prevent recurrence [22]. There are several possible techniques such as trans-jugular intra-hepatic portosystemic stent-shunts, peritoneovenous shunt or percutaneous peritoneal drainage catheters, however there is insufficient evidence to propose one over any other [21]. The same is true for choosing between the use of mesh, primary closure, and even fibrin glue, all of which have been used in various studies. The use of fibrin glue is currently restricted to patients declared unfit/unwilling to undergo operative repair [23]. A recent expert consensus study suggested a decrease in the suitability of mesh repair as the Child's score increases [21].

Ultimately, more evidence is required, and cases should be considered individually, to determine the most effective timing of umbilical hernia repair.

## Consent

Written informed consent was obtained from the patient for publication of this case report and any accompanying images. A copy of the written consent is available for review by the Editor-in-Chief of this journal.

## Competing interests

The authors declare that they have no competing interests.

## Authors' contributions

DWG conceived the manuscript, collected the data, took the photographs, wrote and revised the manuscript. JER collected data and reviewed the manuscript. MS conceived and reviewed the manuscript. PCN wrote the manuscript and performed a final review. EE performed a final review. All authors read and approved the final manuscript.

## References

[B1] Russel RCG Williams NS Bulstrode CJK (eds.) Bailey & Love's Short Practise of Surgery 2008 25 Hodder Arnold21538678

[B2] Park CK The effect of patient positioning on intraabdominal pressure and blood loss in spinal surgery Anesth Analg 2000 91 3 552 557 10960374 10.1097/00000539-200009000-00009

[B3] Cobb WS Burns JM Kercher KW Matthews BD Norton H Heniford BT Normal Intra abdominal Pressure in healthy adults J Surg Res 2005 129 231 235 10.1016/j.jss.2005.06.015 16140336

[B4] Iqbal A Stadlhuber RJ Karu A Corkill S Filipi CJ A study of intragastric and intravesicular pressure changes during rest, coughing, weight lifting, retching and vomiting Surg Endosc 2008 22 12 2571 2575 10.1007/s00464-008-0080-0 18810545

[B5] Sugerman H Windsor A Bessos M Wolfe L Intra-abdominal pressure, saggital abdominal diameter and obesity comorbidity J Intern Med 1997 241 1 71 79 10.1046/j.1365-2796.1997.89104000.x 9042096

[B6] Twardowski ZJ Tully RJ Ersoy FF Dedhia NM Computerized tomography with and without intraperitoneal contrast for determination of intraabdominal fluid distribution and diagnosis of complications in peritoneal dialysis patients ASAIO Trans 1990 36 2 95 103 10.1097/00002480-199004000-00010 2340214

[B7] Papavramidis TS Duros V Michalopoulos A Papadopoulos VN Paramythiotis D Harladtis N Intra-abdominal pressure alterations after large pseudocyst transcutaneous drainage BMC Gastroenterol 2009 9 42 46 10.1186/1471-230X-9-42 19500396 PMC2700125

[B8] Bastani B Dehdashti F Hepatic hydatid disease in Iran, with review of the literature Mt Sinai J Med 1992 62 1 62 69 7739589

[B9] Chao A Chao A Yen YS Huang CH Abdominal compartment syndrome secondary to ovarian mucinous cystadenoma Obstet Gynecol 2004 104 5 Pt 2 1180 1182 15516444 10.1097/01.AOG.0000128106.96563.8b

[B10] Chapman CB Snell AM Roundtree LG Decompensated portal cirrhosis JAMA 1931 97 237 244

[B11] Johnnson JT Ruptured umbilical hernia Trans South Surg Assoc 1901 14 257 268

[B12] Guttormson R Tschirhart J Boysen D Martinson K Are postoperative activity restrictions evidence-based? Am J Surg 2008 195 3 401 403 10.1016/j.amjsurg.2007.12.014 18207126

[B13] Lemmer JH Strodel WE Knol JA Eckhauser FE Management of spontaneous umbilical hernia disruption in the cirrhotic patient Ann Surg 1983 198 1 30 34 10.1097/00000658-198307000-00006 6859990 PMC1352927

[B14] Lalatta Costerbosa G Barazzoni AM Lucchi ML Bortolami R Histochemical types and sizes of fibres in the rectus abdominis muscle of guinea pig:adaptive response to pregnancy Anat Rec 1987 217 1 23 29 10.1002/ar.1092170105 2970237

[B15] Prezant DJ Aldrich TK Karpel JP Lynn RI Adaptation in the diaphragm's in vitro force-length relationship in patients on continuous ambulatory peritoneal dialysis Am Rev Respir Dis 1990 141 5 Pt 1 1342 1349 2339851 10.1164/ajrccm/141.5_Pt_1.1342

[B16] Gilleard WL Brown JM Structure and function of the abdominal muscles in primigravid subjects during pregnancy and the immediate postbirth period Phys Ther 1996 76 7 750 762 8677279 10.1093/ptj/76.7.750

[B17] Kotidis EV Papavramidis TS Ioannidis K Cheva A Lazou T Michalopoulos N Karkavelas G Papavramidis ST The effect of chronically increased intra-abdomial pressure on rectus abdominis muscle histology an experimental study on rabbits J Surg Res 2010 in press 10.1016/j.jss.2010.06.034 20850776

[B18] Baron HC Umbilical hernia secondary to cirrhosis of the liver N Engl J Med 1960 263 824 828 10.1056/NEJM196010272631702 13687191

[B19] O'Hara ET Oliai A Patek AJ Jr Nabseth DC Management of umbilical hernia associated with hepatic cirrhosis and ascites Ann Surg 1973 181 1 85 87 10.1097/00000658-197501000-00018 PMC1343720 1119872

[B20] Granese J Valaulikar G Khan M Hardy H Ruptured umbilical hernia in a case of alcoholic cirrhosis with massive ascites Am Surg 2002 68 8 733 734 12206611

[B21] McKay A Dixon E Bathe O Sutherland F Umbilical hernia repair in the presence of cirrhosis and ascites: results of a survey and review of the literature Hernia 2009 13 5 461 468 10.1007/s10029-009-0535-9 19652907

[B22] Belghiti J Durand F Abdominal wall hernias in the setting of cirrhosis Semin Liver Dis 1997 17 3 219 226 10.1055/s-2007-1007199 9308126

[B23] Melcher ML Lobato RL Wren SM A Novel Technique to Treat Ruptured Umbilical Hernias in Patients with Liver Cirrhosis and Severe Ascites J Laparoendosc Adv Surg Tech A 2003 13 5 331 332 10.1089/109264203769681745 14617394

